# Pathways to adverse pregnancy outcomes: exploring the mediating role of intimate partner violence and depression: results from a South African rape cohort study

**DOI:** 10.1007/s00737-023-01312-5

**Published:** 2023-04-10

**Authors:** N. Abrahams, E. Chirwa, S. Mhlongo, S. Seedat, B. Myers, N. Peer, A. P. Kengne, C. Garcia-Moreno, C. Lombard, R. Jewkes

**Affiliations:** 1 Gender & Health Research Unit, South African Medical Research Council, Francie Van Zijl Dr, Parow Valley, Cape Town, 7501 South Africa; 2 School of Public Health and Family Medicine, Faculty of Health Sciences, University of Cape Town, Anzio Road, Observatory, 7935 Cape Town, South Africa; 3 Office of the Executive Scientist, South African Medical Research Council, Pretoria, 0001 South Africa; 4 Biostatistics Unit, South African Medical Research Council, Francie van Zijl Dr, Parow Valley, 7501 Cape Town, South Africa; 5 Division of Epidemiology and Biostatistics, Department of Global Health, Faculty of Medicine and Health Sciences, Stellenbosch University, Cape Town, South Africa; 6 Non-Communicable Diseases Research Unit, South African Medical Research Council, Francie van Zijl Dr, Parow Valley, 7501 Cape Town, South Africa; 7 Department of Medicine, University of Cape Town, Rondebosch, Cape Town, 7700 South Africa; 8 HRP (The UNDP/UNFPA/UNICEF/WHO/World Bank Special Programme of Research, Development and Research Training in Human Reproduction), Department of Sexual and Reproductive Health and Research, World Health Organization (WHO), 1211 Geneva, Switzerland; 9 Alcohol, Tobacco and Other Drug Research Unit, South African Medical Research Council, Francie van Zijl Dr, Parow Valley, 7501 Cape Town, South Africa; 10 Curtin enAble Institute, Faculty of Health Sciences, Curtin University, Perth, 6102 Australia; 11 Department of Psychiatry and Mental Health, University of Cape Town, Rondebosch, Cape Town, 7700 South Africa; 12 Department of Psychiatry, Faculty of Medicine and Health Sciences, Stellenbosch University, Francie van Zijl Dr, Parow, Cape Town, 7505 South Africa; 13 Department of Psychiatry, Faculty of Medicine and Health Sciences, South African Research Chair in Posttraumatic Stress Disorder, Stellenbosch University, Cape Town, South Africa; 14 Department of Psychiatry, Faculty of Medicine and Health Sciences, Stellenbosch University, Cape Town, South Africa; 15 School of Public Health, Faculty of Health Sciences, University of Witwatersrand, Johannesburg, South Africa

**Keywords:** Intimate partner violence, Adverse pregnancy outcomes, Miscarriage, Abortion, Stillbirths, Mental health, Trauma

## Abstract

Adverse pregnancy outcomes (APOs) are common occurrences that contribute to negative maternal and child health outcomes. Our aim was to test the hypothesis that trauma exposure and depression are drivers of the better-recognised risk factors for miscarriage, abortion and stillbirths. Our comparative cohort study based in Durban, South Africa recruited women who reported a recent rape (*n* = 852) and those who had never experienced rape (*n* = 853), with follow-up for 36 months. We explored APOs (miscarriage, abortion or stillbirth) among those having a pregnancy during follow-up (*n* = 453). Potential mediators were baseline depression, post-traumatic stress symptoms, substance abuse, HbA1C, BMI, hypertension and smoking. A structural equation model (SEM) was used to determine direct and indirect paths to APO. Overall, 26.6% of the women had a pregnancy in the follow-up period and 29.4% ended in an APO, with miscarriage (19.9%) the most common outcome, followed by abortion (6.6%) and stillbirths (2.9%). The SEM showed two direct pathways from exposure to childhood trauma, rape and other trauma, to APO which were ultimately mediated by hypertension and/or BMI, but all paths to BMI were mediated by depression and IPV-mediated pathways from childhood and other trauma to hypertension. Food insecurity mediated a pathway from experiences of trauma in childhood to depression. Our study confirms the important role of trauma exposure, including rape, and depression on APOs, through their impact on hypertension and BMI. It is critical that violence against women and mental health are more systematically addressed in antenatal, pregnancy and postnatal care.

## Introduction

Adverse pregnancy outcomes (APOs) are very common, with estimates that globally 12–22% of pregnancies end in miscarriage (García-Enguídanos et al 2002) and the rate of stillbirth is expected to be 12 per 1000 births by 2030 (Blencowe et al. 2016). The Sub-Saharan African region has among the highest incidences of both miscarriage and stillbirth, contributing a quarter of the total global burden with a substantial impact on both women’s and child’s health (Blencowe et al. 2016). The loss of a pregnancy and a stillborn baby has a substantial lasting impact on both the physical and mental health of women and their families. The health burden is considered higher in regions where such losses are underreported which is often driven by stigma which hampers access to care and support (de Bernis et al. 2016; Garcia-Moreno & Amin 2016). Clinical causes of APOs are well described in the literature (García-Enguídanos et al 2002) and the common biological risk factors for miscarriage include infections, chromosomal abnormalities, hypertension and risk behaviour such as smoking, alcohol use and obesity (Pineles et al. 2014; Ghimire et al. 2020).

Violence against women (VAW) is a pervasive public health and human rights problem affecting one in three women globally, and intimate partner violence (IPV) is the most common form of violence women experience, impacting both morbidity and mortality (Sardinha et al. 2022). The inclusion of a sustainable development goal (SDG) to address gender inequality and empower women (SDG5) through specific targets to eliminate all forms of violence and harmful practices bears testament to the recognition of the impact of VAW on developmental outcomes (Garcia-Moreno & Amin 2016). An extant body of research has shown the impact of such violence on women’s reproductive health with a focus on intimate partner violence (IPV) during pregnancy (Devries et al. 2010; Shamu et al. 2011). An increasing number of reviews, which include studies from low and middle income countries (LMIC), has described the association of IPV and both maternal and neonatal health outcomes (Pallitto et al. 2005, Sarkar 2008, Han and Stewart 2014, Donovan et al. 2016, Hill et al. 2016, Ghazanfarpour et al. 2018, Ahinkorah et al. 2020, Pastor-Moreno et al. 2020) including unintended pregnancies and termination of pregnancies (Pallitto et al. 2013; Hall et al. 2014; Ahinkorah et al. 2020). These studies are largely based on cross-sectional data from Demographic Health Surveys (DHS) or other population-based surveys. Furthermore, these studies are based on self-reported pregnancies and exclude biological assessments such as hypertension, HIV, diabetes and obesity which are well-described risk factors for APO. A review of perinatal health outcomes associated with IPV found these most commonly included fatalities, in the form of perinatal and neonatal deaths and stillbirths, as well as preterm birth and low birth weight (LBW) (Pastor-Moreno et al. 2020). The research has been conducted in different global regions, but there are few papers from Sub-Saharan Africa. A study from Zimbabwe explored both maternal (unplanned pregnancy, late or never booking for antenatal care (ANC), history of miscarriage) and newborn outcomes (neonatal death) and reported associations with IPV experienced in a women’s lifetime, 12 months before pregnancy and during pregnancy (Shamu et al. 2018). In South Africa, an analysis of birth outcomes associated with IPV was conducted using data from a birth cohort in the Western Cape. The first report (*n* = 263) showed an association between past year physical IPV and LBW (Koen et al. 2014); however, a subsequent analysis (*n* = 1137) (Zar et al 2019) did not find any associations between IPV and maternal and child health outcomes rather, food insecurity, smoking and alcohol use during pregnancy were associated with LBW. Such apparent contradictions can occur in analyses in this field as the experience of violence against women intersects with poverty, mental health and alcohol and tobacco use, which are all risk factors for maternal and neonatal health, and these risk factors all intersect with each other (Myers et al. 2018). A study with women in India reported no associations between IPV and APO for the poorest women (Dhar et al. 2018) and the authors note the intersectionality between poverty and reproductive health may play a bigger role in determining the risks for the poorest women in India.

In order to deepen our understanding of the relationship between trauma exposure, particularly violence against women and childhood trauma, recognised risk factors and APOs, we draw on data from the Rape Impact Cohort Evaluation (RICE). We hypothesized that APOs would be influenced by recognised risk factors including hypertension, BMI, smoking and alcohol consumption, as well as prior APOs. However, we additionally hypothesized that these risk factors would be themselves impacted by recognised drivers of these risk factors including poverty, mental health (depression) and prior experiences of trauma. The aim of this paper is to test the hypothesis that trauma experiences and mental health consequences are also drivers of APO (miscarriage, abortion and stillbirths).

## Materials and methods

### Design, setting and population

The Rape Impact Cohort Evaluation (RICE) is a comparative cohort study, its methods are described elsewhere (blinded reference). It was conducted in and around Durban, with participants followed up for between 12 and 36 months. Assessments were conducted every 3 months in the first year and six monthly thereafter. The cohort had two groups of women aged 16–40 years recruited from health services. The one cohort of women who sought care following a recent rape was recruited from post-rape care services (four Thuthuzela Care Centres and a Crisis clinic). The second cohort (control group) of women who had no previous experience of rape was recruited from primary health care services near the post-rape care services.

Women in the post-rape cohort attended the baseline interview within 20 days of the rape. We recruited both HIV-positive and HIV-negative women, but not those who were 14 or more weeks pregnant or breastfeeding. Children under 16 years were excluded, and the upper limit of 40 years was chosen as the study was designed to describe HIV acquisition after the rape and HIV incidence is lower in older women. We recruited from October 2014 to April 2019, and data collection ended in March 2020. We enrolled 852 women who experienced a recent rape and 853 control participants. More details on the study methods are reported elsewhere (blinded reference). Participants were compensated at the recommended basic rate (R80/$5) which increased incrementally at follow-up.

### Assessments

Staff conducted face-to-face interviews and clinical assessments at baseline and follow-up visits in the patient’s preferred language (mainly isiZulu). From 6 months onward, participants were allowed to self-complete parts of the online questionnaire under supervision. Repeated assessments asked participants to report on their experiences in the interval since the last visit. At baseline, we gathered data on age, education employment, relationship status and proxy measures for poverty (income, being a recipient of a child support grant and settlement area, (formal urban, informal urban and rural)) and assessment of food insecurity (how often people in the home go without food (often, sometimes, seldom-hardly ever and never)). We assessed smoking and alcohol use. The latter was assessed using the AUDIT-C which is a sub-scale of the original AUDIT adapted in South Africa (Morojele et al. 2017).

We collected self-reported data on three APO outcomes—miscarriages, abortion and stillbirths—at baseline and follow-up and used the follow-up data in this analysis. For definitions of APO, see Box 1. Clinical assessments were conducted by a professional nurse. A rapid pregnancy test was done using a urine sample. We asked participants at follow-up visits if they were pregnant or if they had an APO in the period between visits. For example, “*Have you had a pregnancy that miscarried since the time of the last interview?*”. We validated pregnancy losses where possible, i.e. when a woman tested positive for pregnancy at one-time point and subsequently tested negative. We tested for HIV infection with two rapid tests and a confirmatory ELISA. Diabetes was diagnosed based on laboratory testing of glycated haemoglobin (HbA1C) levels. A rapid onsite test for *Trichomonas vaginalis* was done using a vaginal swab. Hypertension was determined by blood pressure measurements (three readings were taken, and the average of the 2nd and 3rd readings were used in analysis) and a history of self-reported hypertension (asked if they had ever been told by a health care worker that they have hypertension). Standardized techniques were used to measure heights and weights. Body mass index (BMI) was calculated as weight in kilogrammes divided by height in metres squared (kg/m^2^). We collected data on the number of pregnancies ever, contraceptive use, HIV risk factors, self-reported history if ever having had a sexually transmitted infection (STI), early sexual debut (first sex was at age 15 years or younger) and transactional sexual relationships with a main or casual partner.

**Box 1** Definitions of adverse pregnancy outcomes (APOs)

**Table:** 

Miscarriage: loss of a fetus before 28 weeks of pregnancy
Stillbirth: giving birth to a baby with no signs of life in the third trimester (after 28 weeks of pregnancy)

All mental health measures have been used widely in South Africa. We assessed lifetime trauma using the Life Events Checklist (LEC) (Jewkes et al. 2006, Weathers et al 2013) and childhood adversity using the modified Childhood Trauma Scale (Bernstein et al. 2003, Jewkes et al. 2010). We used the 20-item Centre for Epidemiologic Studies Depression Scale (Radloff 1977; Myer et al. 2008; Nduna et al. 2013) for depression symptomatology and the Davidson Trauma Scale (DTS) for post-traumatic stress symptoms (PTSS) and PTSD (Davidson et al. 1997; Chishinga et al. 2011). We developed binary variables for both using the cut-off for clinical depression (score >  = 16) and the DTS cut-off for probable PTSD diagnosis (score >  = 20) for PTSD frequency. Women were asked about their current relationship status and about lifetime IPV and non-partner sexual violence (NPSV) following the measure from the WHO’s multi-country study on women’s health and violence (Garcia-Moreno et al. 2006). Five items measured physical IPV, seven measured psychological IPV and four measured economic IPV. We did not include in the analysis and assessment of sexual IPV as we screened into the control arm only women who reported no exposure. We combined physical, psychological and economic IPV into a binary variable (none/any (reported 1 or more)), and we derived an additive score to measure the 3 forms of IPV (range 0–16).

### Data analysis

We used STATA 17 for analyses. We enrolled 1705 participants with 852 rape-exposed and 853 in the control group. This secondary data analysis is primarily based on 453 women who had a pregnancy during the follow-up period, i.e. positive pregnancy test at follow-up visits or reported a pregnancy occurred between follow-up visits. The analysis is therefore based on APOs reported in the follow-up period, while exposures are based on data reported at baseline. We excluded 10 women for whom we had no information on the outcome of the pregnancy, i.e. no follow-up data.

We analysed the data descriptively, with categorical data displayed as frequencies with confidence intervals and continuous data displayed as mean and standard deviation (SD). Differences in baseline factors between women in the two arms were assessed using Pearson’s chi-square test or the Student *t*-test. Simple logistic regression was used to assess baseline factors associated with APO. We then built a structural equation model to assess the pathways to APO in the follow-up period using the baseline data. Full information maximum likelihood (FIML) was used in model estimation, and comparative fit index (CFI), Tucker-Lewis index (TLI) and root mean square error of approximation (RMSEA) were used to assess how well the model fitted to observed data. The criteria CFI > 0.95, TLI > 0.90 and RMSEA <  = 0.05 were interpreted as indicative of a good fit. The model included known risk factors for APO (smoking, alcohol use), but we found no direct or indirect pathways and so included these as control variables. We performed a stratified SEM analysis as a sensitivity analysis to assess any differences in pathways to APO between women who reported a rape and women who did not. All statistical tests were done at the 5% significance level.

## Results

At the baseline interview, most of the participants had been previously pregnant (76.7%), and 21.3% reported ever having APOs. In the follow-up period, a quarter of the women (26.6%) had a pregnancy, and 29.4% of these pregnancies ended in an APO, and this did not differ by cohort arm (Table 1). Miscarriage was the most common APO with 19.9% of women reporting a miscarriage. More abortions were reported in the follow-up period than ever (6.6% vs 2.7%). Thirteen stillbirths were reported (2.9% of pregnancies).

**Table 1:** Description of pregnancy, miscarriage, abortion and stillbirths reported at baseline and follow-up period

	*n*/denominator	%	95% confidence intervals	
Reported at baseline *n* = 1307			LCI	UCI
Ever pregnant	1307/1703	76.7	74.6	78.7
Ever miscarriage	206/1307	15.8	13.9	17.8
Ever abortion	36/1307	2.7	1.9	3.7
Ever stillborn	53/1307	4.1	3.1	5.3
Adverse pregnancy outcome	278/1307	21.3	19.1	23.6
Reported at follow-up period *n* = 453				
Pregnant during follow-up	453/1705	26.6	24.5	28.7
Miscarriage during follow-up	90/453	19.9	16.4	23.8
Abortion during follow-up	30/453	6.6	4.7	9.3
Miscarriage and or abortion	120/453	26.5	22.6	30.8
Stillborn during follow-up	13/453	2.9	1.7	4.9
Adverse pregnancy outcome	133/453	29.4	25.3	33.7
Adverse pregnancy outcome among the two cohort groups reported at follow-up				
Women who experienced a rape	61/209	29.2	23.3	35.7
Women who did not experience a rape	72/244	29.5	24.1	35.5

A descriptive analysis of the baseline characteristics of the women who were pregnant during the follow-up period across the cohort groups is shown in Table 2. Women who did not report rape were older, less likely employed and more often dependent on a child support grant than women who experienced rape. While women with rape experience reported higher levels of smoking and alcohol use, they also reported higher levels of IPV, previous trauma and much higher levels of baseline PTSS, depression and suicidal thoughts.

**Table 2:** Descriptive analysis of baseline characteristics among pregnant women by cohort group: women who experienced a rape and women who did not

	*N* = 453	Women who did not experience rape *N* = 244 53.9%	Women who experienced rape *N* = 209 46.1%	*p*-value
Age (mean (SD))	24.6 (24.1, 25.1)	25.2 (24.6, 25.9)	23.8 (23.2, 24.5)	0.003
Education level				
Grade 1–grade 11	42.5 (38, 47.1)	38.7 (32.8, 45)	46.9 (40.2, 53.7)	0.072
Matric/post matric	57.5 (52.9, 62.0)	61.3 (55, 67.2)	53.1 (46.3, 59.8)	
Employed	19.5 (16.1, 23.4)	15.2 (11.2, 20.3)	24.4 (19, 30.7)	0.013
Received a child support grant	36.3 (32, 40.8)	40.7 (34.7, 47.1)	31.1 (25.2, 37.7)	0.029
Relationship status				
No relationship	15.5 (12.4, 19.1)	14 (10.2, 19)	17.2 (12.7, 23)	0.619
Married/living together	7.5 (5.4, 10.4)	8.2 (5.4, 12.4)	6.7 (4, 11)	
Relationship but not living together	77 (72.9, 80.7)	77.8 (72.1, 82.6)	76.1 (69.8, 81.4)	
Settlement area				
Formal township/suburb	70.4 (66.0, 74.4)	77.8 (72.1, 82.6)	61.7 (54.9, 68.1)	0.001
Informal township	16.6 (13.4, 20.3)	12.3 (8.8, 17.1)	21.5 (16.5, 27.6)	
Rural/semi-rural	13.1 (10.2, 16.5)	9.9 (6.7, 14.3)	16.7 (12.3, 22.4)	
Often or sometimes go without food	21.0 (17.5, 25.0)	20.2 (15.6, 25.7)	22 (16.9, 28.2)	0.694
Difficulty to find money	87.2 (83.7, 90.0)	88.5 (83.8, 91.9)	85.6 (80.2, 89.8)	0.361
Trauma & violence experience				
Childhood experiences of trauma (mean)	16.2 (15.9, 16.5)	16.0 (15.6, 16.3)	16.3 (15.9, 16.8)	0.093
Previous trauma (mean)	1.6 (1.4, 1.7)	1.2 (1.0, 1.4)	2.0 (1.8, 2.3)	< 0.001
Any emotional, physical or economic IPV	67.6 (63.1, 71.8)	62.0 (54.4, 69.1)	78.5 (70.5, 84.7)	< 0.001
Health and health behaviour				
Had more than 3 pregnancies	23.6 (19.1, 28.8)	22.3 (16.6, 29.3)	25.4 (18.6, 33.6)	0.373
Previous adverse pregnancy outcome	19.2 (15.8, 23.1)	24.1 (18.2, 31.2)	22.3 (15.9, 30.3)	0.494
Tobacco use	9.1 (6.3, 13.0)	6.6 (3.7, 11.6)	12.3 (7.7, 19.2)	0.014
Alcohol use	52.0 (46.3, 57.7)	50.0 (42.4, 57.6)	54.6 (46.0, 63.0)	0.043
Hazardous alcohol use (audit 4) yes	26.7 (21.9, 32.0)	25.9 (19.8, 33.1)	27.7 (20.7, 36.0)	0.198
HIV positive	49.3 (43.6, 55)	48.2 (40.7, 55.8)	50.8 (42.2, 59.3)	0.697
Positive trichomonas test	4.4 (2.6, 7.4)	4.2 (2, 8.6)	4.6 (2.1, 9.9)	0.302
Age of 1st sex was under 15 years	13.2 (9.8, 17.5)	6.6 (3.7, 11.6)	21.5 (15.3, 29.5)	0.003
Hypertension yes	15.9 (12.1, 20.5)	15.1 (10.4, 21.4)	16.9 (11.4, 24.4)	0.511
BMI (mean)	27.4 (26.8, 28.0)	28.2 (27.4, 29.1)	26.4 (25.6, 27.2)	0.003
HbA1c (mean)	5.3 (5.3, 5.4)	5.3 (5.2, 5.4)	5.3 (5.3, 5.4)	0.991
Mental health				
Post-traumatic stress symptoms (score above 20)	42.6 (37.0, 48.3)	15.1 (10.4, 21.4)	77.7 (69.7, 84.1)	< 0.001
PTSS score (mean)	20.3 (18.5, 22.1)	7.4 (6.1, 8.7)	35.3 (33, 37.7)	< 0.001
Depression (score above 16)	59.8 (54.1, 65.3)	36.7 (29.7, 44.4)	89.2 (82.6, 93.5)	< 0.001
Depression score (mean)	23.1 (21.8, 24.5)	14.4 (13.2, 15.7)	33.2 (31.4, 34.9)	< 0.001

In Table 3, we present the bivariate analysis of socio-demographic, health and trauma experiences and compare those who reported an APO and those who did not in the follow-up period. Few differences were found between the two groups including experiences of rape reported at recruitment. The only significant difference between the two groups was BMI, with women who had an APO having a higher BMI (28.4 vs 26.9: OR 1.03: *p-*value 0.02) compared to women who did not have an APO.

**Table 3:** Bivariate analysis of baseline socio-demographic, trauma, health and mental health characteristics by adverse pregnancy outcome during follow-up

	Adverse pregnancy outcome			
	No (*N* = 320) 70.6 (66.3, 74.7)	Yes (*N* = 133) 29.4 (25.3, 33.7)		*p*-value
	%/mean (95% CI)	%/mean (95% CI)	Crude OR (95% CI)	
Age (mean)	24.6 (24.0, 25.2)	24.6 (23.7, 25.4)	1.00 (0.96, 1.04)	0.998
Education				
Grade 1–grade 11	41.7 (36.4, 47.2)	44.4 (36.1, 52.9)	Ref	
Matric/post matric	58.3 (52.8, 63.6)	55.6 (47.1, 63.9)	0.89 (0.59, 1.34)	0.583
Employed	20.1 (16.0, 24.8)	18.0 (12.4, 25.5)	0.88 (0.52, 1.48)	0.632
Received a child support grant	36.1 (31.0, 41.5)	36.8 (29.1, 45.4)	1.03 (0.67, 1.56)	0.905
Relationship status				
No relationship	14.7 (11.2, 19.1)	17.3 (11.8, 24.7)	Ref	
Married/living together	8.2 (5.6, 11.7)	6.0 (3.0, 11.6)	0.64 (0.25, 1.64)	0.353
Relationship but not living together	77.1 (72.2, 81.4)	76.7 (68.7, 83.1)	0.87 (0.50, 1.50)	0.605
Settlement area				
Formal township/suburb	68.3 (63.0, 73.2)	75.2 (67.1, 81.8)	Ref	
Informal township	16.9 (13.2, 21.5)	15.8 (10.5, 23.0)	0.85 (0.49, 1.48)	0.561
Rural/semi-rural	14.7 (11.2, 19.1)	9.0 (5.2, 15.2)	0.56 (0.28, 1.10)	0.090
Often or sometimes go without food	20.4 (16.3, 25.2)	22.6 (16.2, 30.5)	1.12 (0.69, 1.83)	0.647
Difficult to find money in community when in need	88.7 (84.7, 91.8)	83.5 (76.1, 88.9)	0.64 (0.36, 1.14)	0.127
Violence and trauma experience				
Rape exposed	46.2 (40.8, 51.7)	46.9 (38.5, 55.5)	0.98 (0.66, 1.48)	0.940
Childhood experiences of trauma (mean)	16.0 (15.7, 16.3)	16.6 (16.0, 17.2)	1.06 (1.00, 1.13)	0.052
Previous trauma (mean)	1.5 (1.3, 1.7)	1.8 (1.5, 2.1)	1.11 (0.99, 1.24)	0.076
Any emotional, physical or economic IPV	67.6 (62.3, 72.5)	67.7 (59.2, 75.2)	1.03 (0.67, 1.59)	0.899
IPV score (mean)	6.7 (5.6, 7.8)	7.0 (5.2, 8.9)	1.00 (0.98, 1.02)	0.751
Health and health behaviour				
Had more than 3 pregnancies	25.4 (19.9, 31.8)	19.8 (12.8, 29.3)	0.71 (0.41, 1.22)	0.216
Previous adverse pregnancy outcome	19.4 (15.4, 24.1)	18.8 (13.0, 26.4)	0.96 (0.58, 1.61)	0.887
Tobacco use	9.8 (6.4, 14.7)	7.7 (3.7, 15.3)	0.68 (0.33, 1.43)	0.312
Alcohol use	53.7 (46.8, 60.4)	48.4 (38.3, 58.6)	1.13 (0.75, 1.69)	0.559
Hazardous alcohol use (audit 4)	26.8 (21.2, 33.3)	26.4 (18.3, 36.4)	0.96 (0.60, 1.53)	0.856
HIV positive	51.2 (44.4, 58)	45.1 (35.1, 55.4)	1.21 (0.80, 1.81)	0.366
Positive trichomonas test	2.9 (1.3, 6.4)	7.7 (3.7, 15.3)	1.81 (0.71, 4.64)	0.214
Age of 1st sex was under 15 years	11.7 (8.0, 16.9)	16.5 (10.2, 25.6)	1.75 (0.96, 3.17)	0.066
Transactional sex with main partner	7.8 (4.8, 12.4)	9.9 (5.2, 18)	1.23 (0.61, 2.46)	0.569
Hypertension yes	14.1 (10.0, 19.6)	19.8 (12.8, 29.3)	1.77 (1.02, 3.08)	0.043
BMI (mean)	26.9 (26.2, 27.7)	28.4 (27.3, 29.5)	1.03 (1.00, 1.07)	0.029
HbA1c (mean)	5.3 (5.3, 5.4)	5.3 (5.2, 5.4)	0.82 (0.54, 1.25)	0.362
Mental health				
Post-traumatic stress symptoms (score above 20)	45.4 (38.7, 52.3)	36.3 (27.0, 46.6)	0.87 (0.58, 1.30)	0.493
PTSS score (mean)	20.2 (18.1, 22.3)	20.6 (17.1, 24.2)	1.00 (0.99, 1.01)	0.827
Depression (score above 16)	62.4 (55.6, 68.8)	53.8 (43.5, 63.8)	0.89 (0.59, 1.35)	0.589
Depression score (mean)	22.8 (21.2, 24.4)	23.9 (21.2, 26.6)	1.01 (0.99, 1.02)	0.464
Had suicidal thoughts in last 4 weeks	14.6 (10.4, 20.2)	16.5 (10.2, 25.6)	0.93 (0.53, 1.64)	0.810

**Fig. 1:**
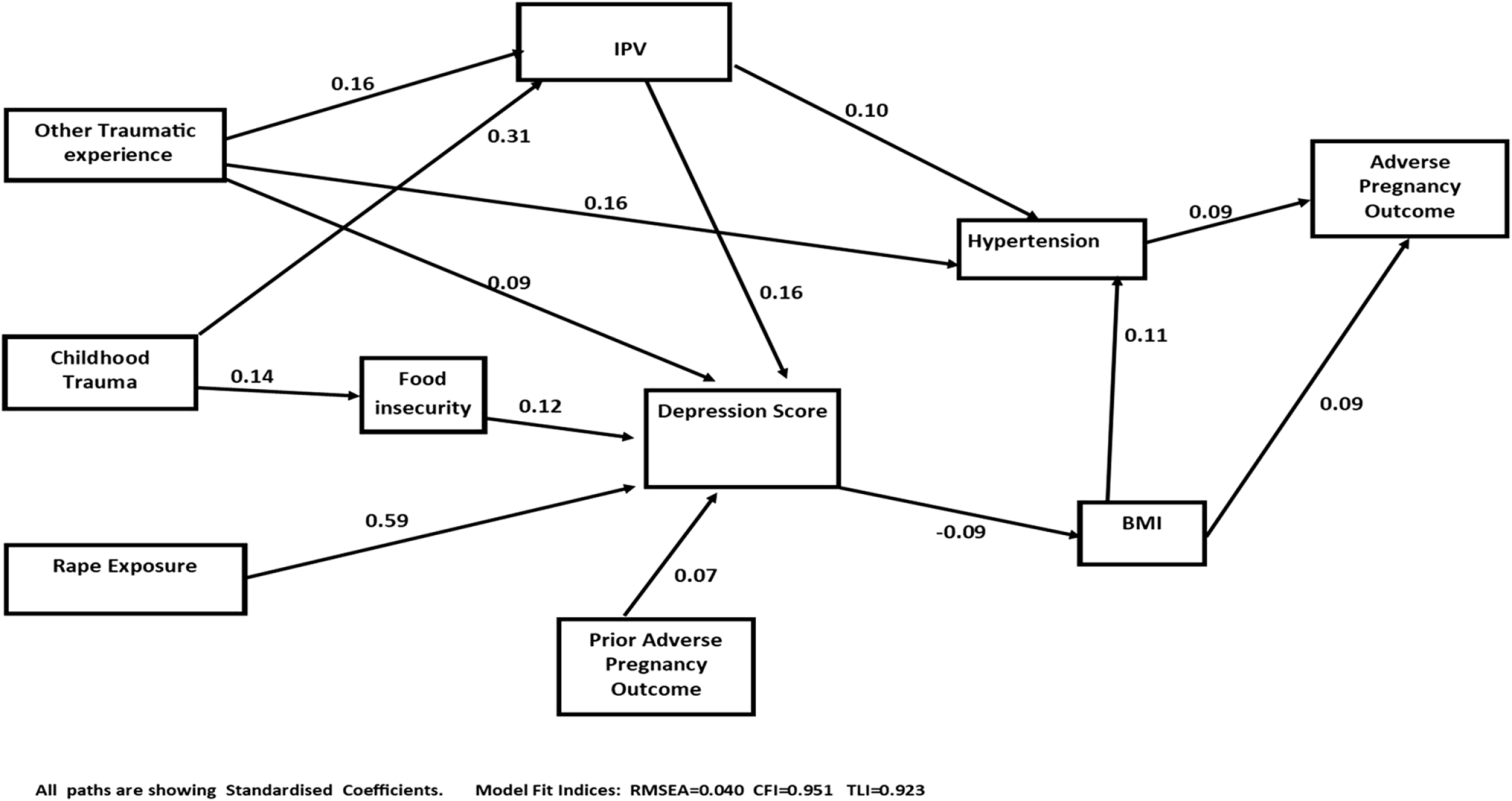
Structural model of pathways to adverse pregnancy outcomes (miscarriage, abortion and stillbirths)

The structural equation model shown in Fig. 1 shows the pathways to APOs, and Table 4 shows the direct, indirect and total effects. Pathways from rape, childhood or other trauma experiences to APO were all mediated by hypertension and/or BMI. Depression also mediated all pathways mediated by BMI, including one from childhood trauma exposure that was also mediated by food insecurity. We found pathways from experiences of trauma in childhood and other trauma to APO that were mediated by intimate partner violence. Paths from intimate partner violence connected to hypertension as well as depression and BMI.

**Table 4:** Model of pathways to adverse pregnancy outcomes (miscarriage, abortion and stillbirths): direct effects, indirect effects and total effects adjusted for smoking and alcohol use

	Direct effect			Indirect effect‡			Total effects		
Path	Standard coef (95% CI)	Unstandardised (95% CI)	*p-*value	Std coef	Unstandardised coef (95% CI)	*p-*value	Std coef	Unstandardised coef (95% CI)	*p-*value
BMI → APO	0.09 (0, 0.19)	0.01 (0, 0.01)	0.047				0.09	0.01 (0.001, 0.014)	0.029
Hypertension → APO	0.09 (− 0.01, 0.18)	0.12 (− 0.01, 0.24)	0.062				0.09	0.12 (− 0.01, 0.24)	0.063
IPV experience → hypertension	0.10 (0.01, 0.20)	0.003(0.001, 0.007)	0.031				0.10	0.003(0.001, 0.007)	0.034
BMI → hypertension	0.11 (0.02, 0.20)	0.01 (0, 0.01)	0.018				0.11	0.01 (0, 0.01)	0.018
Other trauma exposure → hypertension	0.16 (0.06, 0.25)	0.03 (0.01, 0.05)	0.001				0.16	0.03 (0.02, 0.05)	< 0.001
Depression score → BMI	− 0.09 (− 0.18, 0)	− 0.04 (− 0.08, 0)	0.044				− 0.09	− 0.04 (− 0.08, 0)	0.044
Childhood trauma → IPV experience	0.31 (0.23, 0.40)	1.02 (0.72, 1.33)	< 0.001				0.31	1.02 (0.72, 1.33)	< 0.001
Other trauma exposure → IPV experience	0.19 (0.10, 0.28)	1.11 (0.59, 1.63)	< 0.001				0.19	1.11 (0.59, 1.63)	< 0.001
Childhood trauma → food insecurity	0.14 (0.05, 0.23)	0.02 (0.01, 0.03)	0.003				0.14	0.02 (0.01, 0.03)	0.003
IPV experience → depression score	0.16 (0.08, 0.23)	0.22 (0.12, 0.33)	< 0.001				0.16	0.22 (0.12, 0.33)	< 0.001
Food insecurity → depression score	0.12 (0.05, 0.18)	4.11 (1.69, 6.53)	0.001				0.12	4.11 (1.69, 6.53)	0.001
Other trauma exposure → depression score	0.09 (0.02, 0.17)	0.79 (0.18, 1.40)	0.010	0.03	0.25 (0.08, 0.41)	0.003	0.12	1.04 (0.44, 1.64)	0.001
Rape exposure → depression score	0.59 (0.54, 0.65)	17.33 (15.29, 19.37)	< 0.001					17.33 (15.29, 19.37)	< 0.001
Prior APO → depression score	0.07 (0.01, 0.14)	2.73 (0.20, 5.25)	0.034				0.07	2.73 (0.20, 5.25)	0.034
Childhood trauma → depression				0.06	0.3 (0.16, 0.44)	< 0.001	0.06	0.3 (0.16, 0.44)	< 0.001

Indirect effect‡: only significant total indirect effects included in the table

## Discussion

We have shown that APOs, in the form of miscarriage, abortion and stillbirths, were relatively common health problems in our study population. Their prevalence was within the range reported among the general population of pregnant women globally (García-Enguídanos et al 2002). Our data on stillbirths followed global patterns in that they were much less common than miscarriages, although we were not able to calculate a stillbirth rate (Lawn et al. 2005). It is more difficult to compare our data on abortion as different forms of abortion are reported (legal/illegal/spontaneous/induced/incomplete/safe/unsafe) and the denominators are known to be challenging. Our structural equation model confirmed the importance of hypertension and BMI in APOs, which are recognised risk factors for APO, but we did not confirm the role of smoking or harmful alcohol use in the study population.

A key finding was that IPV experiences and multiple trauma experiences, including child abuse and neglect, rape and other traumatic experiences, impacted APOs through their impact on hypertension and on depression and BMI. These pathways suggest stress was the underlying factor, which is further demonstrated by the indirect pathways from trauma experiences and mediated by food insecurity. It is very likely that the impact of these different forms of trauma intersected with each other and was cumulative. Food insecurity and poverty are recognised to increase the risk of experiencing all forms of violence and trauma, as well as poor mental health, although in our model, food insecurity only mediated paths from childhood trauma exposure (Gibbs et al. 2020).

Comparisons with other studies are limited as few studies are based on longitudinal data and definitions of APO are diverse. The only comparison from a Demographic and Health Survey is the prevalence level of APOs reported in the Bangladesh study. The study reported on similar three pregnancy outcomes (miscarriage, abortion and stillbirths) and reported an overall prevalence (ever) of 21% which is similar to the 21.3% reported by our participants at the baseline interview (Afiaz et al. 2020; Halim et al. 2018). Although perinatal mental health is well recognised as a health problem on its own, only one other study has explored mental health indicators of APO, and this was an analysis of 147 pregnant women from a US urban-based cohort which found an increased risk for LBW babies among women who had both IPV experiences and poor mental health (depression and PTSD) (Rosen et al. 2007). While our analysis focused on mental health outside of the pregnancy period, most other studies focus on mental health during the perinatal period, as shown in a systematic review of IPV during pregnancy and mental health in LMIC (Halim et al. 2018). Increasingly, ANC settings are used for identifying women at risk of IPV and linking them to IPV services and IPV prevention interventions ((Pallitto et al. 2016, Kerr-Wilson et al. 2020). In South Africa, reluctance from nursing staff, poor support from management and competing with time for other medical activities in the antenatal period are reported as common obstacles (Cumming et al. 2007). Our findings show that it is important that IPV prevention and assistance for abused women should be regarded as a key part of maternal and child health services to ensure the best maternal and infant outcomes rather than a parallel activity.

The strength of our study is the longitudinal design which allowed us to follow up women with positive pregnancy tests to document the progression, or otherwise, of the pregnancies. This provides confidence in our estimates of early pregnancy loss, particularly as research has shown women often do not recognise early losses, especially if the pregnancy is unplanned. We also had a wide range of both socio-behavioural, and biological risk factors known to contribute to APO (i.e. hypertension, obesity (BMI) and diabetes) and risk factors such as smoking, alcohol use and a previous history of APO. Few abortions were reported in our study. It is possible that even after more than 2 decades of legalisation of abortion in the country, women may still not be keen to report having an abortion (self-induced or through the formal health system), as reported in recent research, because of the continued stigma and shame attached to it (Mavuso and Macleod 2020).

The study limitations include using data reported at baseline on experiences of lifetime IPV and trauma, as well as mental health. We also did not consider these as time-varying covariates in this analysis since we did not collect data on IPV experiences during the pregnancy. It is possible that IPV experiences (and other trauma experiences) in the follow-up period may have had direct effects on APOs. Future studies must explore both lifetime IPV and IPV during pregnancy. Another limitation is that we only included three APOs, as we did not collect data on unintended pregnancies, LBW and prematurity, which are also among the most common APOs associated with IPV. Despite our best efforts, some APOs were self-reported and may have been subject to reporting bias. Also, some women missed visits, and thus the periods between reports and pregnancy tests were not consistently 3 or 6 months, and the longer periods between visits may have impacted on reporting bias. This could have resulted in pregnancies and the loss of the pregnancy not being recorded, resulting in the under-reporting of both pregnancies and APOs. We also may have missed confounders that were not measured. Despite our study limitations, our strength was the robust measures for IPV and the inclusion of both behavioural and biological outcomes including the use of a biological test for pregnancy.

Maternal mental health interventions focus largely on perinatal mental health, commonly postpartum depression, with cognitive behaviour and interpersonal therapy recommended as best practices (Jidong et al. 2021). These interventions do not focus on IPV prevention and are also not feasible in resource-limited settings. Training nurses to deliver a combined IPV and mental health support intervention for pregnant women has shown effectiveness in both the USA (Kiely et al. 2010) and China (Tiwari et al. 2005). A South African ‘Safe and Sound’ intervention adapted the ‘empowerment and counselling’ elements from the USA and Chinese studies with trained professional nurses delivering the intervention in ante-natal clinics. Individual sessions cover building self-efficacy and problem-solving skills and assistance in safety planning (Pallitto et al. 2016). The outcome of this intervention is not yet published. However, it points to the need for further research to combine low-resource mental health support with gender empowerment and transformative interventions. South Africa has been at the forefront of generating evidence on effective gender empowerment intervention for IPV prevention (Kerr-Wilson et al. 2020, Gibbs et al. 2020) and these should be adapted and combined with the World Health Organisation (WHO) developed and tested group-based, lay counsellor facilitated, mental health interventions: Self-Help Plus (SH +) and Problem Management Plus (PM +) which was developed for low-resource setting (Tol et al. 2020). These have been adapted and tested in Uganda (Dawson et al. 2015; Epping-Jordan et al. 2016), Kenya (Bryant et al. 2017) and Pakistan (Rahman et al. 2016) and have shown improvements in mental health indicators 3 and 6 months post-intervention.

## Conclusion

Our analysis has shown that trauma exposure, including intimate partner violence experience, and depression are key drivers of APOs. They are frequently overlooked in studies that consider the drivers of APOs. Thus, addressing the impact of IPV and other trauma exposure in South Africa should be considered central in APO prevention, alongside the biological pathway variables of hypertension and high BMI. Our finding suggests that there may be benefits from IPV prevention interventions and managing depression provided as part of antenatal care. Further research is needed on the benefits of combined IPV and mental health prevention interventions for pregnancy outcomes.

## Data Availability

The data used in this manuscript are available on request to the corresponding author. The main reason is because this longitudinal data set has not fully been analysed by the investigators and students associated with the project.
